# Dietary sources of antioxidants and oxidative stress in age-related macular degeneration

**DOI:** 10.3389/fphar.2024.1442548

**Published:** 2024-09-24

**Authors:** Diana Matías-Pérez, Carlos Francisco Varapizuela-Sánchez, Eduardo Lorenzo Pérez-Campos, Sarahí González-González, Marco Antonio Sánchez-Medina, Iván Antonio García-Montalvo

**Affiliations:** ^1^ División de Estudios de Posgrado e Investigación, Tecnológico Nacional de México/Instituto Tecnológico de Oaxaca, Oaxaca, Mexico; ^2^ Departamento de Ingeniería Química y Bioquímica, Tecnológico Nacional de México/Instituto Tecnológico de Oaxaca, Oaxaca, Mexico; ^3^ Facultad de Medicina y Cirugía, Universidad Autónoma Benito Juárez de Oaxaca, Oaxaca, Mexico

**Keywords:** ROS, vision, AMD, foods, antioxidants

## 1 Introduction

We must consider that as societies experience changes in their eating patterns, there is an increase in the prevalence of chronic noncommunicable diseases (NCDs), which are particularly relevant in older populations and can trigger risk factors for degenerative eye diseases. One of the main issues to highlight is the food transition, which involves a shift towards more Western and processed diets, often rich in refined sugars, saturated fats, and processed foods, thus lacking essential nutrients (antioxidants, vitamins, and minerals) crucial for eye health. Urbanization and fast and convenient food availability have led to increased consumption of unhealthy foods. This not only affects the quality of the diet but can also influence habits such as excessive consumption of alcohol and tobacco, which are risk promoters for the development of ocular pathologies (dry eye, cataracts, age-related macular degeneration, uveitis, among others).

Ocular problems can significantly impact quality of life and increase dependency in those affected (Yang et al., 2019). The current challenge for public health and health professionals is to empower individuals with the knowledge of early detection of visual diagnoses through intraocular pressure measurement and fundus examination, and to emphasize the proactive role of healthy habits (Munteanu et al., 2022). Risk factors associated with ocular pathologies such as glaucoma, age-related macular degeneration (AMD), and cataracts focus not only on age, sex, race, heredity, and cardiovascular health but also on nutritional status (Musa et al., 2023). In the visual process, the retina is the organ responsible for receiving light rays, transforming them into nerve stimuli, and transmitting them through the axons of the optic nerve to the brain, where they are interpreted as vision. The retina covers approximately 2/3 of the back of the inner surface of the eyeball (Mahabadi et al., 2024).

The adult retina is composed of ten differentiable layers corresponding to 4 cell layers: the retinal pigment epithelium (RPE), the photoreceptors, the intermediate connecting neurons, and the ganglion cells, which give rise to the optic nerve. The other layers correspond to synaptic connections (nerve cells and limiting membranes) (Mahabadi et al., 2024). The impact of a balanced diet rich in antioxidants on these different layers of the retina makes studies of their efficacy significant in various pathological processes of the eye (Dziedziak et al., 2021). AMD is one of the most common pathologies in the population and is the main cause of severe vision loss in both eyes in people over 60 years of age (Thomas et al., 2021). The macula is a small region of the posterior retina, approximately 5–6 mm in diameter. Within it is the macula lutea, rich in the xanthophyll carotenoids (Lutein and Zeaxanthin), which possess antioxidant properties and protect against blue light damage. In addition, the macula contains a central depression called the fovea, designed to provide maximum visual resolution (Arunkumar et al., 2020).

AMD can present in two fundoscopic forms: the dry or nonexudative atrophic form and the neovascular or exudative form, also known as the wet form (Fernandes et al., 2022). The dry form of AMD is the most common morphologic variant and can progress to the wet or neovascular form. In the latter, choroidal neovascular membranes in the central region of the retina can lead to hemorrhages, exudation, and significant loss of vision. Neovascular membranes result from abnormal vascular growth or angiogenesis induced by the release of vascular endothelial growth factor (VEGF). In the late form of AMD, the “dry” or nonexudative type is characterized by the development of geographic atrophy or atrophic scars in the macula. Although some patients with atrophic AMD do not show significant improvement with standard doses of anti-VEGF, adjusting the frequency of injections to their needs may help maintain vision stability and prevent further deterioration. In the absence of signs of disease activity, the dosing interval can be gradually increased, and if the disease reactivates, it can be shortened. Unfortunately, treatment options for “dry” AMD or geographic atrophy are currently minimal. There is no definitive cure for this condition, but some investigational treatments aim to slow the progression of the disease. Some preventive measures that can help include quitting smoking and adopting a diet rich in antioxidants.

AMD is a disease that affects the macula lutea, a small region of the retina responsible for central vision. One of the most common symptoms of AMD is drusen. These deposits can interfere with vision, causing problems such as blurred vision, distortion of images, and the perception of a fixed black spot in the center of the visual field. It can also make it difficult to distinguish colors and adapt to changes in light. In the wet form of AMD, abnormal blood vessels develop, which can lead to more severe complications (Gheorghe et al., 2015). The appearance of drusen and the growth of these vessels result from chronic changes in the macula, affecting structures such as the retinal pigment epithelium and Bruch’s membrane (Bhutto and Lutty, 2012). Drusen are classified into two types: hard and soft. Hard drusen are smaller, usually smaller than 63 μm, while soft drusen are larger, exceeding 125 μm (Pollreisz et al., 2021). Histologically, hard drusen are accumulations of hyaline material, whereas soft drusen are clusters of hard drusen (Spaide and Curcio, 2010). AMD can present in two forms: the dry form, which is the most common and can progress to the wet form, and the wet form, which can cause hemorrhages and significant vision loss (Fernandes et al., 2022). The latter is related to abnormal blood vessel growth driven by VEGF. In the advanced stages of AMD, the dry form is characterized by atrophic scar formation in the macula (Fleckenstein et al., 2021; Thomas et al., 2021). Although there is no definitive cure for this disease, there are treatments under investigation that seek to slow its progression. Currently, treatment options for the dry form are limited. However, taking preventive measures like quitting smoking and following an antioxidant-rich diet can benefit eye health.

AMD primarily affects central vision, essential for daily tasks such as reading, driving, and recognizing faces. Late stages of wet and dry AMD are generally associated with severe visual loss, profoundly affecting overall quality of life (Waugh et al., 2018; Gehrs et al., 2006). AMD is a multifactorial disease that involves an interaction between genetic and environmental factors (Cascella et al., 2014). Other risk factors include smoking, uncontrolled blood pressure, and lack of a diet rich in fruits and green leafy vegetables (Shetty et al., 2023; Boeing et al., 2012). Some treatments, such as anti-VEGF drugs, can help save vision in the wet form of AMD (Vogel et al., 2017). However, there is currently no 100% effective treatment; what is sought with its implementation is to prevent severe visual loss.

Although aging is one of the main risk factors for the onset of AMD, and although this pathology is multifactorial, oxidative stress must also be considered; oxidative stress causes damage to different types of ocular cells, contributing significantly to the development of this disease. This article highlights the challenge of nutritional intervention, both due to the regulatory environment and the complexities in designing clinical trials to address these issues. It is crucial to modify certain environmental factors, such as smoking cessation and the adoption of a healthy diet, to prevent or slow the progression of AMD. Until now, no recommendations for daily nutritional supplementation as a preventive method for AMD in healthy individuals have been established. However, for those with some degree of the disease, supplements such as AREDS (Vitamins C and E, Beta-carotene, Zinc and Copper), AREDS2 (Vitamins C and E, Lutein, Zeaxanthin, Omega-3 acids and Zinc), and an increased intake of additional nutrients (carotenoids, resveratrol, and omega-3 fatty acids) are recommended to promote eye health in the population. Antioxidants play a crucial role in cell defense against oxidative stress, which is essential to prevent the progression of AMD and preserve patients’ vision; consuming nutrients with high antioxidant capacity is emerging as a promising strategy for treating this disease. Antioxidant-rich foods have been described in more detail below.

## 2 Pathogenesis and oxidative stress of AMD

The relationship between oxidative stress and AMD is direct; due to its oxygen-rich environment and exposure to light, the retina is prone to forming free radicals. To mitigate the damage caused by these radicals, there are enzymatic and non-enzymatic mechanisms that maintain homeostasis (Wang et al., 2022). However, in the aging retina, antioxidant systems are attenuated, resulting in oxidative damage that manifests itself in changes such as hard exudates in the periphery, thickening of Bruch’s membrane, and thinning of the choriocapillaris (Kushwah et al., 2023). Oxidative stress induces the accumulation of lipid and complement deposits between the RPE and Bruch membrane, leading to the thickening of the Bruch membrane, an early pathological feature of AMD. In addition, oxidative stress causes RPE dysfunction and death, critical for photoreceptor support and function, leading to progressive vision loss in this pathology (Tisi et al., 2021).

Although these changes are natural to ocular aging, other risk and pathophysiological factors are activated to cause the pathology. The most common are genetic susceptibility, microglial activation, complement system stimulation, loss of homeostasis between pro-inflammatory and anti-inflammatory factors, and macrophage polarization in individuals with risk factors for the developing the pathology (Wendimu and Hooks, 2022). Oxidative stress induces a chronic inflammatory response, with activation of the complement system and macrophage recruitment; this sustained inflammation exacerbates retinal cell damage and accelerates the progression of AMD (Abokyi et al., 2020).

Histopathology of the retina in patients with AMD shows that this ocular disease is distinguished by selective and focal destruction of the retinal layers in the macular region, which is crucial for central vision. Retinal findings in individuals with AMD are varied, although they share certain characteristic features. These include shrinkage of the RPE and photoreceptor layer, accumulation of lipid and protein deposits beneath the RPE or in Bruch’s membrane, as well as choriocapillary atrophy, choroidal neovascularization, and disciform scar formation (Abokyi et al., 2020; Natoli et al., 2017; Gupta et al., 2003).

In addition to structural changes, AMD is also characterized by an inflammatory response, with the recruitment of macrophages and microglial cells, as well as the activation of the complement system (Abokyi et al., 2020). In its natural location, the RPE performs several vital functions for the balance of the retina, such as regulating the transport of nutrients and metabolites, absorbing light, recycling the visual pigment essential for continuous phototransduction, and phagocytizing the outer segments of photoreceptors that are shed (Hurley, 2021). A molecular event associated with the malfunction of RPE in AMD is the accumulation of lipofuscin. Lipofuscin is a remnant of the outer segments of photoreceptors that have been poorly degraded and phagocytosed by RPE. This accumulation of lipofuscin in RPE can contribute to oxidative damage through the generation of free radicals and inhibit the phagocytic degradation of damaged biomolecules and organelles (Różanowska, 2023).

The retina has a high metabolic rate and oxygen consumption, creating an environment conducive to forming reactive oxygen species (ROS). The retina contains high levels of polyunsaturated fatty acids (PUFA), which are highly reactive and can contribute to ROS generation. Furthermore, the retina contains photosensitive molecules, such as rhodopsin and lipofuscin, which can contribute to ROS formation through photochemical reactions. Finally, exposure to light can also induce oxidative stress in the retina by generating ROS through photochemical reactions involving rhodopsin and other photosensitive molecules. As these physiological conditions combine, they create an environment ripe for the developing oxidative stress in the retina (Beatty et al., 2000). PUFAs are highly susceptible to ROS oxidation, which generates lipid radicals and lipid peroxidation products such as reactive aldehydes (4-hydroxyalkenals, malondialdehyde, acrolein) (Kodali et al., 2020). PUFA and photosensitive molecules can induce ROS production through various mechanisms, such as lipid peroxidation, photosensitization, activation of ROS-producing enzymes (NADPH oxidases), and mitochondrial dysfunction (Juan et al., 2021); these processes feed on each other and perpetuate oxidative stress in ocular cells. Another source of oxidative stress associated with AMD is smoking. Cigarette smoke extract has been shown to significantly increase lipid peroxidation *in vitro* RPE by up to eight times compared to controls (Kunchithapautham et al., 2014).

Regarding the genetic factor, at least 14 genes have been associated with AMD (Stradiotto et al., 2022). The gene that has shown the strongest association with AMD is complement factor H (*CFH*) (Toomey et al., 2018); it is a glycoprotein with the function of maintaining a balanced immune response by modulating complement activation (Parente et al., 2017). Several studies have demonstrated the presence of complement cascade proteins in drusen, suggesting that inflammation may play an important role in AMD (Armento et al., 2021). Currently, rare variants have been identified in protein-coding regions of the *CFI*, *C3*, *C9*, *TIMP3,* and *SLC16A8* genes in AMD, establishing the role of the complement pathway in the pathogenesis of AMD (den Hollander et al., 2022). Following the active phase of AMD-GWAS research between 2010 and 2016, strategies were emphasized to evaluate AMD loci functionally. Of the 16 new AMD loci reported in 2016, 13 have not been further analyzed (Strunz et al., 2020). This could be attributed in part to small effect sizes and possibly to a preferential location of the associated variants in noncoding regions of the genome.

Another essential factor to consider is obesity, especially abdominal obesity, and a sedentary lifestyle since high abdominal circumference values have a twice as high risk of developing AMD (Zhang et al., 2016). Increased body weight can have adverse health effects, including increased oxidative stress, increased risk of chronic inflammation, and an imbalance in blood lipid levels; this lipid imbalance is related to the pathogenic mechanisms of AMD (Haas et al., 2015). Previous research has shown that excess body fat can influence carotenoid transport and deposition processes from the blood to the macula, ultimately leading to a decrease in the level of macular pigment in the fovea (Bovier et al., 2013).

## 3 Functional foods and supplements used in the preventive treatment of age-related macular degeneration

In a healthy state, the body employs a variety of enzymatic and nonenzymatic antioxidants to protect cells from the damaging effects of ROS. These antioxidants work to neutralize ROS and prevent cell damage and death. However, the cellular capacity to counteract ROS accumulation becomes compromised under pathological conditions, such as in AMD. This occurs due to a combination of factors - a decrease in the efficiency of antioxidant systems, reduced production of ROS scavengers and other protective molecules, and an overproduction of ROS itself. As a result, the delicate balance between ROS generation and antioxidant defenses is disrupted, allowing oxidative stress to take hold and contribute to the development and progression of AMD (Chaudhary et al., 2023; Tyuryaeva and Lyublinskaya, 2023).

In recent years, the National Eye Institute (USA) has conducted two significant multicenter studies, known as AREDS and AREDS2, which have significantly advanced our scientific understanding of AMD treatment. These studies evaluated the safety and efficacy of antioxidant supplements and other nutrients in treating AMD. Results show that supplementation with Lutein, minerals, omega-3 fatty acids, and fatty acid derivatives likely slows progression to late AMD (Chew et al., 2009; Age-Related Eye Disease Study 2 Research Group, 2013; Chew et al., 2022; Keenan et al., 2024). However, the minimum effective dose of an individual antioxidant and whether a combination of components is necessary to achieve an optimal formulation has not yet been determined. However, zinc-rich supplements are recommended (Age-Related Eye Disease Study Research Group, 1999; AREDS2 Research Group et al., 2012). The diet intakes of vitamins, carotenoids, essential fatty acids, and other trace elements have been investigated for their potential protection against age-related eye diseases. The results of studies related to the nutritional effects of these dietary factors on AMD are presented and discussed below (see Table 1).

**TABLE 1:** Foods with antioxidant potential.

Antioxidant molecule	Food source	Participation in AMD pathology	Recommended doses	References
Lutein and Zeaxanthin	Parsley, spinach, kale, and egg yolk	Protect the macula from the damaging effects of blue light	10 mg of lutein and 2 mg of zeaxanthin per day for at least 6 months	Mrowicka et al. (2022)
Crocetin and norbixin	Saffron and achiote	Regulate oxidative stress	0.6 mg of Crocetin per day, while norbixin is 0.3 mg/kg body weight per day for 3 months	Camelo et al. (2020)
Zinc	Beans, lima beans, oysters, meat, seafood, fish, poultry, cereals, and grains	By acting as a co-factor for antioxidant enzymes, this substance helps to enhance their ability to neutralize and eliminate harmful free radicals and other reactive oxygen species. This, in turn, provides protection against the detrimental effects of oxidative damage to cells, tissues, and biomolecules	25 mg per day for 6 months	Gilbert et al., 2019; Mrowicka et al., 2022
Vitamin C	Citrus fruits, berries, and red pepper	Reduces oxidative stress, promotes immune protection, and reduces the probability of neovascular AMD.	500 mg per day for 6 months	Khoo et al., 2019; Saini et al., 2022; Mrowicka et al., 2022
Omega-3	Nuts and seeds, plant oils, nuts, and fish	Reduces inflammation, decreases oxidative stress, and promotes photoreceptor membrane structure	EPA (3.4 g) and EPA (1.6 g) per day for at least 6 months	Querques and Souied, 2014; Fan and Song, 2022; Prokopiou et al., 2019; Perumal et al., 2022; Carneiro and Andrade, 2017
Resveratrol	Red grapes, wine, and berries	Increases antioxidant enzyme levels and activates the transcription factor Nrf2	150–450 mg daily for 3 months	Dziedziak et al., 2021; Lin et al., 2016

Lutein and Zeaxanthin are abundant water-soluble carotenoids in foods such as parsley, spinach, kale and egg yolk. These compounds offer multiple health benefits, acting as antioxidants by scavenging reactive oxygen species (ROS) and forming complexes with essential proteins in the human body (Mrowicka et al., 2022; Abdel-Aal et al., 2013). Carotenoids are diverse natural pigments synthesized in plants, algae, and some bacteria. They all share a standard structure: a tetraterpenoid backbone of forty carbon atoms organized into eight isoprene units. They can be classified into two main categories: carotenes and xanthophylls (Snodderly, 1995). Carotenes are unsaturated hydrocarbons, meaning they comprise only carbon and hydrogen atoms.

The absorption of carotenoids from the diet is a complex process that depends on several factors. During digestion, these compounds are released, and their bioavailability increases. How food is prepared is crucial; for example, cooking food breaks down cell walls, facilitating the release of carotenoids (Molteni et al., 2022). In addition, the presence of fats in food is essential, as carotenoids are incorporated into particles called chylomicrons, which transport lipids into the bloodstream (Hussain, 2014). Physiological factors, such as digestive health and the composition of the gut microbiome, also play a role (Rocha et al., 2023). On the other hand, a high fiber intake can limit the absorption of fats and, thus, carotenoids (Palafox-Carlos et al., 2011). Xanthophylls, such as Lutein and Zeaxanthin, are oxygenated carotenoids with oxygen atoms (Snodderly, 1995; Abdel-Aal et al., 2013). These carotenoids are essential for ocular health and are not precursors of vitamin A, so they are called non-provitamin A carotenoid (Abdel-Aal et al., 2013). Other macular carotenoids of interest include mesozeaxanthin, β-cryptoxanthin, capsanthin, astaxanthin, and fucoxanthin.

Lutein and Zeaxanthin, crucial carotenoids found in the retina, particularly in the macula, are essential for ocular health and visual function. Their primary role is to shield the macula from the harmful effects of blue light (Mrowicka et al., 2022), a high-energy light that can induce oxidative stress and contribute to the onset of AMD and other ocular conditions (Cougnard-Gregoire et al., 2023). These carotenoids act as natural sunscreens, absorbing and blocking detrimental blue light before it reaches the delicate cells of the retina. In addition to their light-filtering role, Lutein and Zeaxanthin have been proven to enhance visual acuity, sharpness, and clarity of vision. They also serve as potent antioxidants, aiding in the removal of harmful radiation that can harm retinal cells and exacerbate eye diseases (Roberts and Dennison, 2015). Including Lutein- and Zeaxanthin-rich foods in the diet, or taking supplements, can help maintain healthy levels in the macula and promote overall eye health (Graydon et al., 2012). A daily dose of 10 mg of Lutein and 2 mg of Zeaxanthin for at least 6 months is recommended to help slow the progression of AMD (Mrowicka et al., 2022).

In addition to the carotenoids mentioned above, others, such as crocetin and norbixin can also be used to regulate oxidative stress. Saffron components, such as crocin and crocetin, benefit patients with AMD. In *in vitro* studies, saffron extracts and their main components have shown neuroprotective actions, and crocin protects photoreceptors against cell death induced by intense blue or white light illumination. Norbixin, a water-soluble natural dye obtained from the seeds of the achiote shrub, has been shown *in vitro* assays to significantly reduce VEGF production and several inflammatory cytokines, such as IL-6 and IL-8, leading to a reduction in ROS (Camelo et al., 2020). The recommended intake of crocetin is 0.6 mg per day, while norbixin is 0.3 mg/kg body weight per day for 3 months to delay the progression of AMD (Camelo et al., 2020). Regulatory agencies such as the European Union (EU) have approved health claims on carotenoids, including those used to regulate oxidative stress. According to Regulation (EC) number 1924/2006, these claims are backed by scientific evidence and comply with established regulations, providing a reassuring framework for their use.

Beans and lima beans are significant sources of zinc, a crucial nutrient involved in the structure and function of various protein complexes. Zinc is the second most abundant transition metal in the human body, with approximately 2 g present, mostly within cells (Blasiak et al., 2020). Free zinc is an essential component in cells; however, its levels in this state are low due to accumulation toxicity, and it can be found in organelles such as the endoplasmic reticulum, the Golgi apparatus, and mitochondria (Costa et al., 2023). Zinc is distributed in well-regulated gradients relative to the plasma membrane and intracellular compartments (Bafaro et al., 2017).

It is chemically compatible with several ligands present on the histidine, aspartate, glutamate, and cysteine residues of many proteins. Unlike other transition metals, zinc is an inert redox metal, which plays an important role in regulating enzymes involved in oxidative processes. It also facilitates the deprotonation of water by decreasing its pKa, making it an excellent enzyme cofactor. Most of the zinc in the human body is found in skeletal muscle and bone mass, accounting for approximately 60% and 30%, respectively (Thompson, 2022). The eye has a relatively high content of zinc, particularly in the RPE, where it is stored mainly in intracellular compartments in ganglion cells, horizontal cells, amacrine cells, and Müller cells of the retina. Additionally, zinc can be present in photoreceptor outer segments (POSs), degraded by RPE cells. As endogenous zinc is co-released with glutamate from the synaptic terminals of photoreceptors, it may help protect the retina from glutamate toxicity (Gilbert et al., 2019). The established dose of Zinc consumption is 25 mg per day for 6 months (Mrowicka et al., 2022).

Citrus fruits such as oranges and grapefruit are rich in vitamin C, which is a potent antioxidant that protects various biomolecules such as proteins, lipids, carbohydrates, and nucleic acids from damage caused by free radicals and ROS (Saini et al., 2022). Vitamin C has been shown to reduce the probability of neovascular AMD (Khoo et al., 2019). The established dose of vitamin C consumption is 500 mg per day for 6 months (Mrowicka et al., 2022).

Foods rich in antioxidants and omega-3 fatty acids, such as nuts, fish, walnuts, and seeds, may play an essential role in preventing and treating AMD (Querques and Souied, 2014). The mechanism of action of omega-3 fatty acids in AMD is mainly based on their anti-inflammatory and anti-angiogenic properties. Inflammation is a critical process in the pathogenesis of AMD, as it contributes to the destruction of retinal cells and the pigment epithelium (Fan and Song, 2022). Omega-3 fatty acids, such as eicosapentaenoic acid (EPA) and docosahexaenoic acid (DHA), have anti-inflammatory effects that may help reduce inflammation in the retina and prevent cell destruction. Omega-3 fatty acids have improved retinal function in animal models (Prokopiou et al., 2019). This is achieved by increasing the optical density of macular pigment, which enhances light sensitivity and visual acuity (Perumal et al., 2022). The recommended omega-3 intake is 3.4 g of DHA and 1.6 g of EPA per day for at least 6 months (Carneiro and Andrade, 2017).

Resveratrol is a phytophenol found in red grapes, wine, and berries. This compound is part of the antifungal defense mechanisms of plants and has been shown to offer beneficial effects in animal organisms. Among its properties, resveratrol is notable for its ability to prevent coronary heart disease and its antioxidant, anti-inflammatory, anti-aging, and anticarcinogenic activity. It is worth mentioning that resveratrol protects against oxidative stress by acting on reactive oxygen species (superoxide, hydroxyl, and hydrogen peroxide), activating modulators of gene transcription (*SIRT1*), and influencing NADPH oxidase. In human RPE cell cultures exposed to hydrogen peroxide, resveratrol has been shown to increase antioxidant enzyme levels and reduce ROS production (Dziedziak et al., 2021). The recommended consumption dose is 150–450 mg daily for 3 months (Lin et al., 2016).

We believe that the response to antioxidants and their interaction with drugs can vary significantly between patients due to genetic, environmental, and lifestyle factors, making it difficult to establish predictors of these interactions on an individual basis. Although some interaction mechanisms have been described *in vitro* or animal models, clinical evidence on the therapeutic implications of these interactions in patients with AMD is limited.

In addition to the above, it should be noted that the food and nutritional transition that is taking place globally poses significant challenges since the increase in the consumption of processed foods, rich in saturated fats and simple sugars, which are cheaper than healthy foods, directly affects low-income populations, increasing malnutrition due to deficiency and excess, which leads to the appearance of chronic diseases that will lead to risk factors for AMD. On the other hand, public policies that address environmental pollution and the proper treatment of urban solid waste are required since pollutants can exacerbate the inflammatory responses associated with AMD.

## 4 Conclusion

In conclusion, AMD is a devastating eye disease that affects millions of people worldwide, especially those of advanced age. This condition is characterized by the gradual accumulation of lipid and protein deposits in the retina, leading to atrophy of the RPE and, in some cases, to the formation of neovascular membranes that can cause hemorrhages and loss of vision. One of the main factors contributing to the development and progression of AMD is oxidative stress. Due to its high metabolic rate, high oxygen consumption, and the presence of polyunsaturated fats and photosensitive molecules, the retina is particularly vulnerable to this type of stress. Free radicals generated by oxidative stress can damage retinal cells and accelerate degeneration. To reduce the risk of developing AMD or slow its progression, healthy and balanced diet rich in natural foods containing antioxidants, such as brightly colored fruits and vegetables, should be adopted. The consumption of processed and ultra-processed foods should be reduced, as well as the intake of alcohol and tobacco. Despite these advances, research on factors such as lipid metabolism and the chronic inflammatory process should continue to understand the pathogenesis of this disease better and develop more effective prevention and treatment strategies.
